# High prevalence of diabetes among young First Nations Peoples with metabolic dysfunction-associated steatotic liver disease: a population-based study in Australia

**DOI:** 10.1186/s12939-024-02153-z

**Published:** 2024-04-30

**Authors:** Patricia C. Valery, Shruti Roche, Catherine Brown, James O’Beirne, Gunter Hartel, Barbara Leggett, Richard Skoien, Elizabeth E. Powell

**Affiliations:** 1 Cancer & Chronic Disease Research Group, QIMR Berghofer Medical Research Institute, 300 Herston Road, 4006 Herston, QLD Australia; 2 The University of Queensland, St Lucia, QLD Australia; 3 Department of Gastroenterology and Hepatology, Royal Brisbane and Women’s Hospital, Brisbane, QLD Australia; 4 Department of Gastroenterology and Hepatology, Sunshine Coast University Hospital, Sunshine Coast, QLD Australia; 5 School of Nursing, Queensland University of Technology, Brisbane, QLD Australia; 6 Department of Gastroenterology and Hepatology, Princess Alexandra Hospital, Brisbane, QLD Australia; 7 Centre for Liver Disease Research, Translational Research Institute, Faculty of Medicine, The University of Queensland, St Lucia, QLD Australia

**Keywords:** Type 2 diabetes, Cirrhosis, Population, Epidemiology, NAFLD

## Abstract

**Background:**

Liver disease is an important contributor to the mortality gap between First Nations Peoples and non-Indigenous Australian adults. Despite a high burden of metabolic comorbidities among First Nations Peoples, data about the epidemiology of metabolic dysfunction-associated steatotic liver disease (MASLD) in this population is scarce.

**Methods:**

A retrospective analysis of all adults hospitalized with MASLD or metabolic dysfunction-associated steatohepatitis (MASH) with/without cirrhosis during 2007–2019 in the state of Queensland was performed. Patients were followed from the first admission with MASLD/MASH (identified based on validated algorithms) to decompensated cirrhosis and overall mortality. We explored differences according to Indigenous status using Multivariable Cox regression.

**Findings:**

439 First Nations Peoples and 7,547 non-Indigenous Australians were followed for a median of 4.6 years (interquartile range 2.7–7.2). Overall, women were overrepresented, but more so in the First Nations cohort (72.7% vs. 57.0%, *p* < 0.001). First Nations patients were younger, a higher proportion lived in remote and socioeconomic disadvantaged areas, and had higher comorbidity compared to non-Indigenous Australians (all *p* < 0.001). Diabetes, the most common comorbidity affecting both groups, was overrepresented in First Nations Peoples versus non-Indigenous Australians (43.5% vs. 30.8%, *p* < 0.001, respectively). Nineteen (4.3%) First Nations Peoples and 332 (4.4%) of non-Indigenous patients progressed to cirrhosis decompensation (9.0% [95%CI 4.5–17.7] vs. 7.7% [95%CI 6.6–8.9; *p* = 0.956] respectively within 10 years). In multivariable analysis, there was no association between Indigenous status and progression to decompensated cirrhosis (*p* = 0.759) and survival (*p* = 0.437).

**Conclusions:**

This study provides the first population-based epidemiological data on MASLD in First Nations Australians. The high prevalence of diabetes (that is associated with advanced fibrosis and liver disease mortality) among young First Nations Peoples with MASLD raises concern about future risk of progressive liver disease in this patient population. These data highlight the importance of early identification of MASLD, and providing culturally appropriate intervention to reduce disease progression in parallel with the management of cardiometabolic comorbidities.

**Supplementary Information:**

The online version contains supplementary material available at 10.1186/s12939-024-02153-z.

## Background

Liver disease is an important contributor to the mortality gap between Aboriginal and Torres Strait Islander peoples, the First Nations Peoples of Australia, and non-Indigenous Australian adults. Liver cancer is the third most common cancer-related death in First Nations men, and incidence rates are approximately double that of non-Indigenous Australians [1].

In Queensland (2007–2016), the third-most populous Australian state, the rate of cirrhosis hospitalisation was 3.4-times higher for First Nations Peoples compared to non-Indigenous Australians [2]. Moreover, unadjusted survival was poorer for First Nations Peoples hospitalised for cirrhosis [3] and for those diagnosed with hepatocellular carcinoma (HCC) [4], with part of the survival deficit explained by sociodemographic and clinical factors including comorbidity burden.

Alcohol misuse, chronic hepatitis B and C, and metabolic dysfunction-associated steatotic liver disease (MASLD) are important causes of cirrhosis among all Australians [2, 5]. First Nations Peoples experience a disproportionately higher burden of alcohol misuse, and chronic hepatitis B and C infection compared to non-Indigenous Australians [6–8]. However, data about the epidemiology of MASLD in First Nations Peoples is scarce. In a population-based linkage study of cirrhosis admissions which included 779 First Nations Peoples, 23 Indigenous patients (3.0%) had MASLD or metabolic dysfunction-associated steatohepatitis (MASH) compared to 5% of non-Indigenous Australians (*p* = 0.005) [3]. Among all patients with cirrhosis, the prevalence of alcohol-related cirrhosis and hepatitis C virus remained stable during 2007–2016, that of MASLD/MASH increased by 67% (*p* < 0.001), and the prevalence of diabetes mellitus nearly doubled (from 13.7 to 25.4%; *p* < 0.001) [5].

Diabetes mellitus is associated with an increased risk of advanced fibrosis, cirrhosis-related complications, and liver disease mortality [9]. Obesity, lipid abnormalities, and hypertension are also important risk factors for severe liver disease [9]. In a population-based linkage study of people hospitalized with MASLD or MASH with or without cirrhosis in the state of Queensland, 37.1% of the patients with MASLD-related cirrhosis and diabetes mellitus progressed to decompensated cirrhosis within a decade [10]. These risk factors, in particular including diabetes and obesity, are overrepresented in First Nations Peoples versus non-Indigenous Australians [6]. The primary aim of this study was to compare the sociodemographic and clinical characteristics (in particular the prevalence of comorbidity) in First Nations Peoples and non-Indigenous Australians with MASLD/MASH admitted to a Queensland hospital during 2009–2018. The secondary aim included exploratory analyses to examine differences in patient outcomes (cumulative incidence of decompensated cirrhosis and overall mortality) according to indigenous status.

## Methods

We conducted a population-based retrospective cohort study of people hospitalized with MASLD/MASH with or without cirrhosis in the state of Queensland, Australia. The details of the study have been previously described [10]. Briefly, the primary data source for this study included the Queensland Hospital Admitted Patient Data Collection that comprises information on all hospital episodes of care for patients admitted to Queensland public and private hospitals, and the Death Registry Data. The source population included all adult patients (≥ 20 years) with at least one hospital admission in Queensland during July-2007 to Dec-2019 with a recorded diagnosis of MASLD/MASH. We excluded patients whose age was < 20 years, residential location was unknown, and those who did not reside in Queensland. Following the global expert consensus statement that recommended the use of currently available ICD codes for NAFLD/NASH to define MASLD and MASH [11], a case of MASLD/MASH was defined by at least one hospitalization with any of the following ICD-10-AM codes: NAFLD (K76.0), NASH (K75.8) or other and unspecified cirrhosis of liver (K74.6). As in other NAFLD/MASLD linkage studies [12], patients who ever had other liver diseases recorded in a hospitalization (e.g. alcohol-related liver disease, viral hepatitis, autoimmune liver disease, hemochromatosis, Wilson’s disease), or ICD-10-AM codes associated with alcohol use disorder or somatic consequences of alcohol use were excluded [12].

A case of metabolic dysfunction-associated fatty liver disease (MASLD) or metabolic dysfunction-associated steatohepatitis (MASH) was defined by at least one hospitalization with an ICD-10-AM code for NAFLD (K76.0), NASH (K75.8) or other and unspecified cirrhosis of liver (K74.6). As described by Hagstrom et al. [12] and Petta et al. [13] patients who ever had other liver diseases recorded in a hospitalization were excluded. The complete list of exclusions comprised: alcoholic liver disease, viral hepatitis, autoimmune liver disease, hemochromatosis, Wilson’s disease, Alpha-1-antitrypsin deficiency, Budd-Chiari syndrome, chronic hepatitis, unspecified, secondary/unspecified biliary cirrhosis, or ICD-10-AM codes associated with alcohol use disorder or somatic consequences of alcohol use (e.g. F10 Mental and behavioural disorders due to use of alcohol, K70 Alcoholic liver disease, T51 Toxic effect of alcohol, K86.0 Alcohol-induced chronic pancreatitis) [12]. We selected the first hospital admission with MASLD/MASH during Jul-2009 to Dec-2018 (referred to as index admission), discharged alive and survived for at least 30 days. We excluded patients who had a code for liver decompensation, a history of liver transplant or HCC prior to the index admission. All patients had a minimum look-back period of 2 years and follow-up period of 1 year. As median survival of patients with decompensated cirrhosis is approximately 2 years, a two-year look-back period will likely identify the first admission for decompensation for most cases. The follow up period of 1 year is adequate to capture sufficient outcome events (death, hospital admissions) [14].

### Measurements

Sociodemographic data obtained from the Queensland Hospital Admitted Patient Data Collection (QHAPDC) included: age group at admission (e.g. 20–24 years, 25–29 years,… to 75 + years), gender, marital status, country of birth, place of residence, and Indigenous status (patients were coded as First Nations Peoples if identified in at least one of their records within the study period). Area-based measures of remoteness of residence [15] and socioeconomic status [16] were based on place of residence. Comorbidity at the time of hospital admission was measured using the Charlson Comorbidity Index (CCI) [17]. All diseases listed in the CCI as primary or other diagnosis were analysed (excluding liver disease) using validated coding algorithms [18].

The data includes all admitted patient separations from public hospitals and private hospitals in the state of Queensland. When patients were transferred within the same hospital (e.g. from the emergency department to a ward) or to another hospital, we considered these episodes of care as one ‘hospital stay’. Hospital sector for each ‘hospital stay’ was categorized as ‘public hospital only’ or ‘private only or mix’.

Patients were followed from the first hospital admission with MASLD/MASH with or without cirrhosis. The primary outcomes were progression to decompensated cirrhosis identified by the first decompensation event (having an ICD-10-AM code for ascites, hepatic encephalopathy, or oesophageal variceal bleeding) and overall mortality.

### Data analysis

All statistical analyses were performed in Stata 18.0 (StataCorp). Descriptive analyses are presented as frequency (percentages, %) or median (interquartile range, IQR) value depending on data distribution. All p-values are 2-sided. The cumulative incidence of decompensated cirrhosis and mortality (death of any cause) were explored using the Kaplan–Meier method (log-rank statistic). For the former, patients were followed from the index admission to date of decompensation event or were censored at date of death (any cause), liver transplant, HCC diagnosis, or 31 December 2019. For the latter, all cases were followed until date of death (of any cause) or were censored at date of liver transplant, HCC diagnosis, or Dec-2019. Multivariable Cox regression analysis was used to assess the differences in cumulative incidence of decompensated cirrhosis and mortality according to selected sociodemographic and clinical characteristics. Hazard ratios (HRs) with associated 95% confidence intervals (CIs) were reported. The final multivariable models were based on the results of the bivariable analysis considering our understanding of the relationships and dependencies among variables as well as their clinical relevance. A Least Absolute Shrinkage and Selection Operators (LASSO) penalised regression cox proportional hazards model was used to identify a parsimonious model including variables that had the strongest association with the outcomes (decompensated cirrhosis and mortality) [19]. Variables included in the models were checked to ensure that they adhered to the assumption of proportional hazards over time (Schoenfeld residuals). The vce(robust) option was used to obtain robust standard errors for the parameter estimates to control for mild violation of underlying assumptions. Multivariable logistic regression analysis with extrahepatic cancers as dependent variable (presence vs. not) was used to examine the difference in prevalence of extrahepatic cancers at index admission according to Indigenous status adjusting for age group.

The study was approved by the Metro South Health Services and QIMR Berghofer Human Research Ethics Committees (HREC/17/QPAH/23; P2209).

## Results

**Fig. 1:**
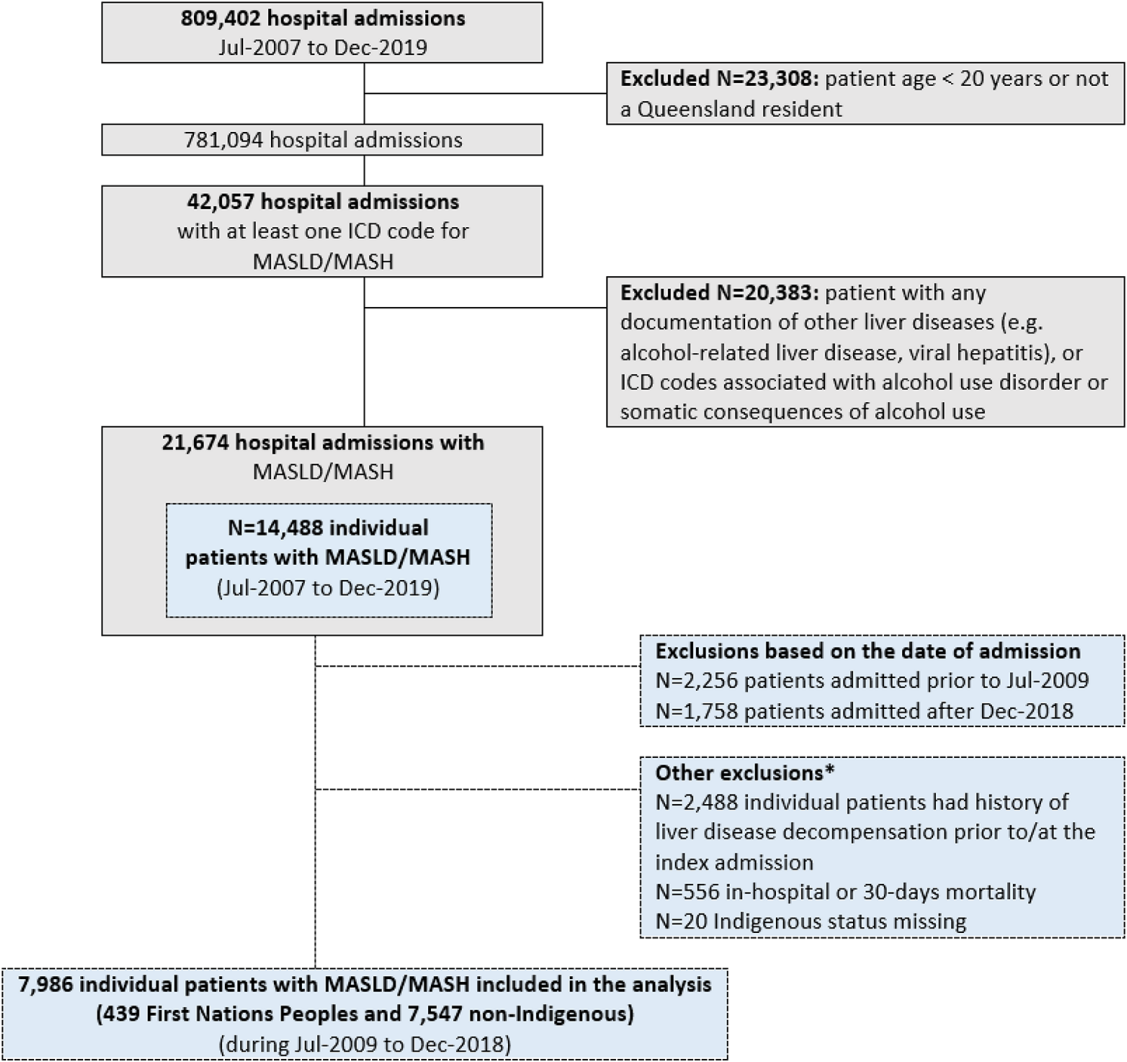
Flowchart for case ascertainment. *Note* Metabolic dysfunction-associated steatotic liver disease (MASLD); Metabolic dysfunction-associated steatohepatitis (MASH); International Classification of Diseases 10th edition– Australian Modification (ICD-10‐AM). *Patients may have more than one exclusion criteria

During Jul-2007 and Dec-2019, there were 42,057 hospitalizations with a recorded ICD-10-AM code for NAFLD/NASH. After exclusions described in Fig. 1, a total of 7,986 individual patients were included in the analysis: 439 (5.5%) were identified as First Nations Peoples, and 7,547 (94.5%) as non-Indigenous Australians. The proportion of First Nations Peoples in the study cohort did not vary during Jul-2009 and Dec-2018 (*p* = 0.31). Patients were followed for a median of 4.6 years (interquartile range (IQR) 2.7–7.2) with no significant difference according to Indigenous status (4.2 years (IQR 2.6–6.8) for First Nations Peoples vs. 4.6 years (IQR 2.7–7.2) for non-Indigenous Australians, *p* = 0.14).

**Fig. 2:**
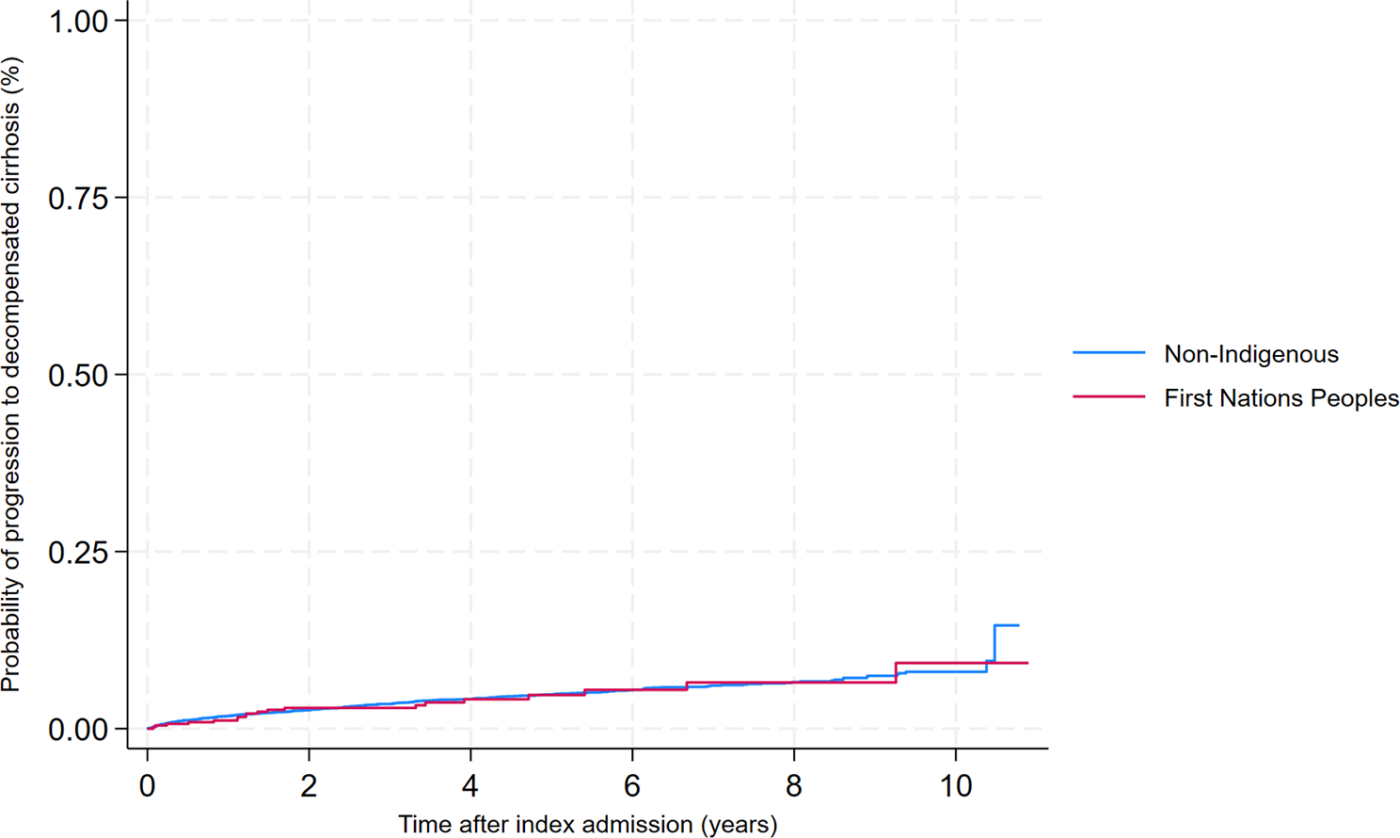
Cumulative incidence of decompensated cirrhosis according to Indigenous status (Kaplan–Meier analysis)

The characteristics of the cohort of patients categorized according to Indigenous status is described in Table 1. Women were overrepresented in both groups, but more so in the First Nations cohort (72.7% vs. 57% of non-Indigenous patients, *p* < 0.001). About half of the First Nations patients were younger than 50 years (51.5% vs. 33.6% of non-Indigenous patients, *p* < 0.001) and had no partner (51.3% vs. 39.0%, *p* < 0.001), and about two-thirds lived outside major city areas (64.0% vs. 39.5%, *p* < 0.001) and in most disadvantaged areas (66.5% in quintiles Q4 and Q5 vs. 44.1%, *p* < 0.001). First Nations and non-Indigenous patients were similar with regard to the prevalence of cirrhosis at index admission (13.2% vs. 13.9%, respectively, *p* = 0.70).

**Table 1:** Patient sociodemographic and clinical characteristics at index hospital admission with MASLD/MASH with or without cirrhosis according to indigenous status

		Non-Indigenous	First Nations Peoples	*p*-value
		*N* = 7,547 (%)	*N* = 439 (%)	
**Sociodemographic factors**				
Sex	Male	3,243 (43.0%)	120 (27.3%)	**< 0.001**
	Female	4,304 (57.0%)	319 (72.7%)	
Age group	20–39 years	1,265 (16.8%)	115 (26.2%)	**< 0.001**
	40–49 years	1,266 (16.8%)	111 (25.3%)	
	50–59 years	1,769 (23.4%)	103 (23.5%)	
	60–69 years	1,734 (23.0%)	78 (17.8%)	
	70 years and over	1,513 (20.0%)	32 (7.3%)	
Marital status^a^	Married/De Facto	3,866 (61.0%)	183 (48.7%)	**< 0.001**
	No partner	2,470 (39.0%)	193 (51.3%)	
Rurality of residence	Major city	4,561 (60.4%)	158 (36.0%)	**< 0.001**
	Inner regional	1,609 (21.3%)	93 (21.2%)	
	Outer regional	1,226 (16.2%)	119 (27.1%)	
	Remote/very remote	151 (2.0%)	69 (15.7%)	
Socioeconomic status	Q1 most affluent	1,378 (18.3%)	37 (8.4%)	**< 0.001**
	Q2	1,477 (19.6%)	44 (10.0%)	
	Q3	1,365 (18.1%)	66 (15.0%)	
	Q4	1,564 (20.7%)	122 (27.8%)	
	Q5 most disadvantaged	1,763 (23.4%)	170 (38.7%)	
Hospital sector	Public	3,840 (50.9%)	321 (73.1%)	**< 0.001**
	Private or mix	3,707 (49.1%)	118 (26.9%)	
**Clinical factors at index admission**				
Cirrhosis		1,047 (13.9%)	58 (13.2%)	0.70
Portal hypertension		154 (2.0%)	5 (1.1%)	0.19
Charlson comorbidity groups	CCI = 0	4,479 (59.3%)	214 (48.7%)	**< 0.001**
	CCI = 1	597 (7.9%)	35 (8.0%)	
	CCI = 2	1,406 (18.6%)	103 (23.5%)	
	CCI = 3	1,065 (14.1%)	87 (19.8%)	
Diabetes		2,321 (30.8%)	191 (43.5%)	**< 0.001**
Obesity		2,072 (27.5%)	145 (33.0%)	**0.011**
Hypertension		1,020 (13.5%)	65 (14.8%)	0.44
Major adverse cardiovascular events^b^		631 (8.4%)	32 (7.3%)	0.43
Renal disease		319 (4.2%)	30 (6.8%)	**0.009**
Chronic pulmonary disease		248 (3.3%)	17 (3.9%)	0.50
Extrahepatic cancers^d^		318 (4.2%)	8 (1.8%)	**0.014** ^**e**^
Disorders of lipoprotein metabolism		269 (3.6%)	21 (4.8%)	0.18
Mood disorders		134 (1.8%)	< 1.0%^**c**^	0.25^**f**^
Anxiety disorders		154 (2.0%)	6 (1.4%)	0.33

*Note MASLD* Metabolic dysfunction-associated steatotic liver disease, *MASH* Metabolic dysfunction-associated steatohepatitis, *CCI* Charlson Comorbidity Index

Bold values indicates statistically significant (*p* < 0.05)

^**a**^Information missing for *N* = 1,274 (16.0%)

^**b**^Major adverse cardiovascular event included four items from the Charlson comorbidity index, namely: myocardial infarction, congestive heart failure, peripheral vascular disease, cerebrovascular disease

^c^Fisher’s exact test

^**d**^Extrahepatic cancers include any cancer except hepatocellular carcinoma

^**e**^In multivariable analysis adjusting for age group *p* = 0.114

^f^Exact numbers not reported due to privacy issues

### Comorbidities

First Nations Peoples had a significantly higher burden of comorbidity; 51.3% had at least one comorbidity listed in the Charlson index compared to 40.7% for non-Indigenous Australians (*p* < 0.001), 43.5% had diabetes (vs. 30.8%, *p* < 0.001), 33.0% had obesity (vs. 27.5%, *p* = 0.011), and 6.8% had chronic renal disease (vs. 4.2%, *p* = 0.009). While fewer First Nations Peoples had extrahepatic cancers at index admission (1.8% vs. 4.2%, *p* = 0.014) vs. non-Indigenous Australians, in multivariable analysis adjusting for age group this difference was not statistically significant (*p* = 0.114).

### Incidence of decompensated cirrhosis

At the end of the follow-up period 19 (4.3%) First Nations Peoples progressed to cirrhosis decompensation compared to 332 (4.4%) of non-Indigenous patients. Among those who progressed to cirrhosis decompensation, the median time from index admission to the first episode of decompensation was 1.4 years for First Nations Peoples (IQR 0.8–3.9) vs. 1.5 years (IQR 0.4–3.3) for non-Indigenous Australians. The average proportion of First Nations Peoples progressing to decompensated cirrhosis was 1.0% per year (95%CI 0.6–1.5), and 9.0% (95%CI 4.5–17.7) had at least one admission with cirrhosis complications within 10 years compared to 1.0% per year (95%CI 0.9–1.1) and 7.7% (95%CI 6.6–8.9), respectively, for non-Indigenous Australians (*p* = 0.956). See Kaplan Meier curves for the overall cohort (Fig. 2), and according to sex and age group (Supplementary Fig. 1). These similarities were reflected in the unadjusted hazard rates comparing First Nations Peoples with non-Indigenous Australians (HR = 0.99, 95%CI 0.62–1.57). In multivariable analysis, when adjusting for age group only, First Nations Peoples were 34% more likely to progress to decompensated cirrhosis (adj-HR = 1.34, 95%CI 0.84–2.13), but chance could not be ruled out. Adding cirrhosis and portal hypertension (adj-HR = 1.03, 95%CI 0.65–1.63), and comorbidities (diabetes, cancer, and hypertension - adj-HR = 0.91, 95%CI 0.58–1.44), one at a time, did not alter the hazard ratio substantially. In the final adjusted model including all the abovementioned variables, there was no association between Indigenous status and progression to decompensated cirrhosis (adjusted HR = 1.08, 95%CI 0.68–1.71).

**Fig. 3:**
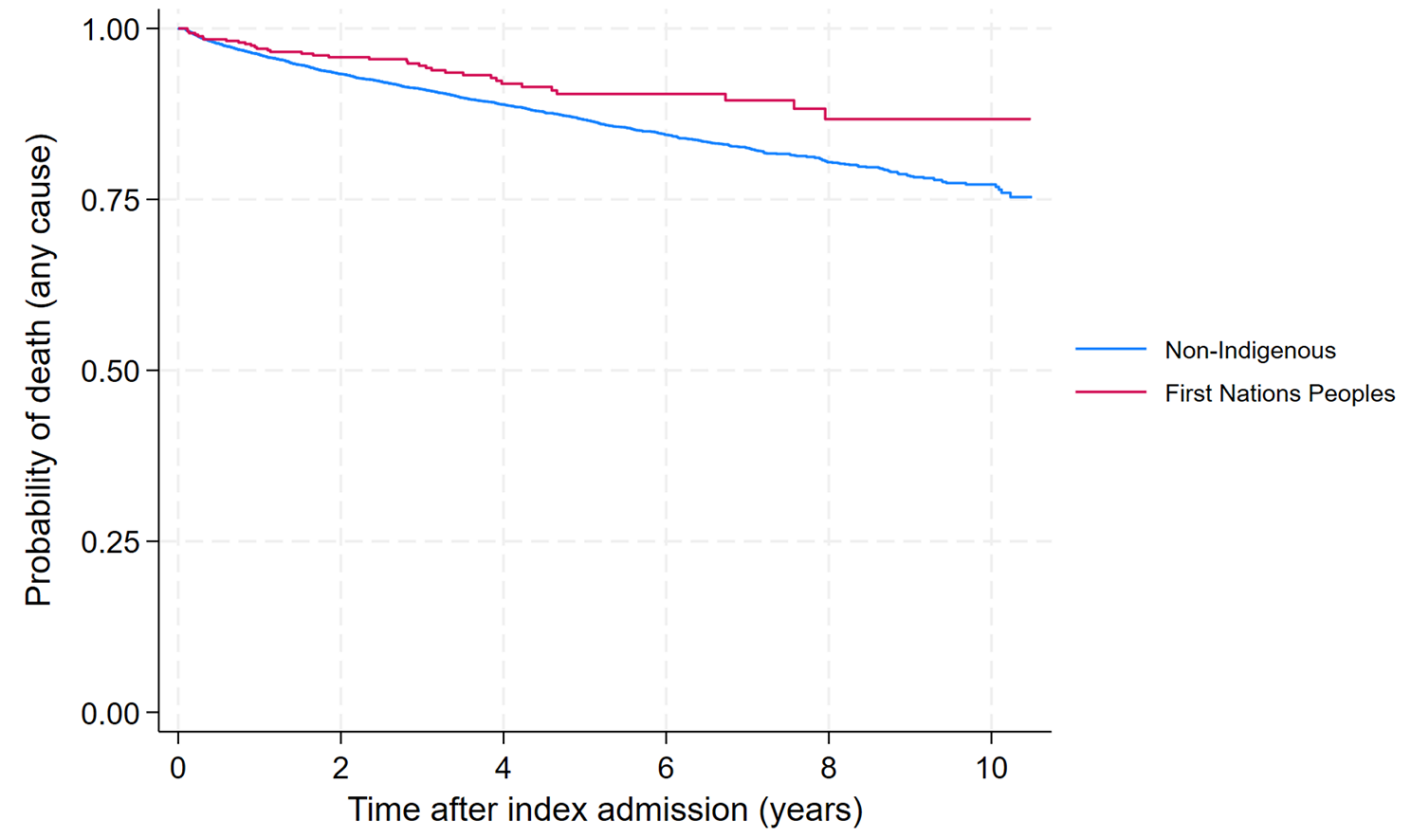
Kaplan Meier survival curve for overall mortality according to indigenous status

### Survival

At the end of the follow-up period 35 (8.0%) First Nations Peoples had died compared to 990 (13.1%) of non-Indigenous patients. During the follow-up period, 50 (0.6%) non-Indigenous patients were diagnosed with HCC and eight had a liver transplant (0.1%), and no First Nations Peoples had either HCC or liver transplant.

The probability of 10-year survival for First Nations Peoples was 86.7% (95%CI 80.5–91.1) versus 77.2% (95%CI 75.4–78.9; *p* = 0.005), as demonstrated in Kaplan Meier curves for the overall cohort (Fig. 3), and according to sex and age group (Supplementary Fig. 2). In univariable analysis, First Nations Peoples were 39% less likely to die within the follow-up period compared to non-Indigenous Australian (HR = 0.61, 95%CI 0.44–0.86; *p* = 0.005) (Table 2). In multivariable analysis, the disparity in survival according to Indigenous status was mostly explained by differences in age. When adjusting for age group only, the association was no longer significant (adjusted HR = 0.99, 95%CI 0.69–1.49; *p* = 0.950). Adding the other sociodemographic variables (sex, socioeconomic status and hospital sector - adj-HR = 0.57, 95%CI 0.40–0.80), cirrhosis and portal hypertension (adj-HR = 0.63, 95%CI 0.45–0.88), and comorbidities (diabetes, obesity, major cardiovascular events, renal disease, chronic pulmonary disease, cancer, disorders of lipoprotein metabolism, and mood disorders - adj-HR = 0.61, 95%CI 0.43–0.86), one at a time, did not alter the hazard ratio substantially. In the final adjusted model including all the abovementioned variables, there was no significant difference in survival between First Nations and non-Indigenous patients (adj-HR = 0.87, 95%CI 0.60–1.24, *p* = 0.437).

**Table 2:** Cox regression analysis of factors associated with progression to decompensated cirrhosis and overall mortality among 7,986 patients admitted with MASLD/MASH with or without cirrhosis

		Progression to decompensated cirrhosis		Overall mortality	
		Unadjusted HR (95%CI)	Adjusted^b^ HR (95%CI)	Unadjusted HR (95%CI)	Adjusted^c^ HR (95%CI)
Indigenous status	First Nations Peoples (vs. Non-Indigenous)	0.99 (0.62–1.57)	1.08 (0.68–1.71)^e^	**0.61 (0.44–0.86)**	0.87 (0.60–1.24)^f^
**Sociodemographic factors**					
Sex	Female (vs. Male)	**0.74 (0.60–0.91)**	N/S	**0.71 (0.62–0.80)**	0.95 (0.83–1.09)
Age group	20–39 years	Reference	Reference	Reference	Reference
	40–49 years	1.44 (0.75–2.78)	1.20 (0.63–2.29)	**2.40 (1.46–3.94)**	**2.15 (1.30–3.56)**
	50–59 years	**3.93 (2.27–6.80)**	**2.52 (1.45–4.37)**	**3.91 (2.48–6.16)**	**3.24 (2.03–5.15)**
	60–69 years	**6.35 (3.72–10.85)**	**3.06 (1.76–5.32)**	**9.01 (5.83–13.93**)	**5.94 (3.78–9.34)**
	70 years and over	**8.45 (4.94–14.44)**	**3.15 (1.78–5.55)**	**29.88 (19.52–45.74)**	**13.86 (8.83–21.76)**
Marital status	No partner (vs. Married/De Facto)	1.04 (0.83–1.31)	N/S	**1.53 (1.35–1.74)**	**1.22 (1.06–1.40)**
Rurality of residence	Major city	Reference	N/S	Reference	N/S
	Inner regional	1.28 (1.00-1.63)		1.09 (0.94–1.27)	
	Outer regional	0.84 (0.61–1.16)		0.97 (0.82–1.16)	
	Remote/very remote	0.32 (0.10-1.00)		0.63 (0.39–1.02)	
Socioeconomic status	Q1 most affluent	Reference	N/S	Reference	Reference
	Q2	1.41 (0.99–2.01)		1.13 (0.91–1.41)	1.14 (0.88–1.48)
	Q3	1.07 (0.73–1.57)		1.18 (0.95–1.46)	1.17 (0.92–1.50)
	Q4	1.31 (0.92–1.87)		**1.36 (1.11–1.67)**	**1.28 (1.01–1.63)**
	Q5 most disadvantaged	1.39 (0.99–1.96)		**1.41 (1.16–1.72)**	1.15 (0.90–1.46)
Hospital sector	Private or mix (vs. Public)	**0.76 (0.62–0.94)**	N/S	**0.68 (0.60–0.77)**	**0.75 (0.65–0.86)**
**Clinical factors at index admission**					
Cirrhosis		**10.84 (8.77–13.39)**	**6.94 (5.40–8.90)**	**4.00 (3.51–4.56)**	**1.87 (1.59–2.19)**
Portal hypertension		**9.31 (6.68–12.96)**	**2.20 (1.51–3.21)**	**4.40 (3.40–5.69)**	**1.65 (1.19–2.31)**
Diabetes		**2.72 (2.21–3.36)**	**1.97 (1.58–2.47)**	**2.02 (1.79–2.29)**	**1.29 (1.12–1.48)**
Obesity		0.80 (0.62–1.04)	N/S	**0.53 (0.45–0.63)**	**0.74 (0.61–0.90)**
Hypertension		1.11 (0.84–1.49)	0.79 (0.58–1.08)	**2.33 (2.03–2.66)**	N/S
Major adverse cardiovascular events^a^		**1.59 (1.13–2.22)**	N/S	**4.82 (4.19–5.54)**	**2.55 (2.17–3.01)**
Renal disease		**1.88 (1.23–2.88)**	N/S	**5.22 (4.41–6.18)**	**1.80 (1.47–2.21)**
Chronic pulmonary disease		1.19 (0.68–2.07)	N/S	**2.93 (2.35–3.64)**	**1.46 (1.16–1.85)**
Extrahepatic cancers^d^		**4.14 (2.98–5.76)**	**2.49 (1.71–3.63)**	**6.62 (5.54–7.91)**	**4.60 (3.71–5.70)**
Disorders of lipoprotein metabolism		1.22 (0.77–1.94)	N/S	**1.58 (1.24–2.01)**	1.07 (0.79–1.44)
Mood disorders		1.01 (0.45–2.25)	N/S	**1.67 (1.16–2.41)**	**1.72 (1.17–2.55)**
Anxiety disorders		1.19 (0.58–2.43)	N/S	1.17 (0.77–1.77)	N/S

*Note N/S* Variable not selected for inclusion in the final model, *MASLD* Metabolic dysfunction-associated steatotic liver disease, *MASH* Metabolic dysfunction-associated steatohepatitis, *HR* Hazard ratio, *CI* confidence interval, *CCI* Charlson Comorbidity Index

Bold values indicates statistically significant (*p* < 0.05)

^**a**^Major adverse cardiovascular event included four items from the Charlson comorbidity index, namely: myocardial infarction, congestive heart failure, peripheral vascular disease, cerebrovascular disease

^**b**^Final model included Indigenous status, age group, cirrhosis, portal hypertension, diabetes, hypertension, and extrahepatic cancer

^**c**^Final model included Indigenous status, age group, sex, socioeconomic status, hospital sector, cirrhosis, portal hypertension, diabetes, obesity, major cardiovascular events, renal disease, chronic pulmonary disease, extrahepatic cancer, disorders of lipoprotein metabolism, and mood disorders

^**d**^Extrahepatic cancers include any cancer except hepatocellular carcinoma

^e^*p* = 0.759

^f^*p* = 0.437

## Discussion

This study provides the first population-based epidemiological data concerning MASLD/MASH in a large cohort of an indigenous population in a developed country. In comparison to non-Indigenous Australians with MASLD/MASH, the most striking differences were that First Nations Peoples were predominantly female, substantially younger, and had a higher burden of concurrent conditions.

Australia is a highly developed country, and First Nations Peoples, like indigenous populations in comparable countries, are over-represented in low socioeconomic strata and have poorer access to health services [20]. However, comparing our findings with that of indigenous groups in similar countries is not possible, as comparable data about MASLD/NAFLD in these groups are also lacking. Nevertheless, there are reports that the prevalence of clinical findings suggestive of MASLD in many indigenous populations may be similar to the global prevalence (e.g. indigenous Arctic populations [21], Maori and Pacific peoples in New Zealand) [22], and that MASLD-related mortality is comparable to their non-indigenous counterparts (e.g. American Indians and Alaska Natives in the US experienced similar rising trend in NAFLD-related mortality during 1999–2022 compared to white individuals) [23].

In this study, diabetes was the most common comorbidity recorded at admission and was present at a disproportionately higher rate in First Nations Peoples with MASLD/MASH than in non-Indigenous patients. This finding is consistent with previous reports showing a higher burden of disease secondary to diabetes in all Indigenous populations around the globe. Australian First Nations People appear to have the greatest burden of this complication and are 2.9 times more likely to develop diabetes than their non-Indigenous counterparts, and at a significantly premature age of onset [20, 24]. Additionally they tend to have worse glycaemic control and higher rates of diabetes-related complications, contributing to higher rates of potentially preventable hospitalisations [4, 5]. Females are disproportionately affected with a higher prevalence of both impaired glucose tolerance and diabetes than Indigenous Australian males [1]. The reasons driving the prevalence of diabetes in Indigenous Australians are complex. Higher rates of cigarette smoking and obesity in this group certainly contribute, as well as lower socioeconomic status and the social determinants of health [6, 7], factors that also contribute to development of MASLD. There is growing evidence for intrauterine exposures on the later development of chronic diseases, with maternal pre-gestational and gestational diabetes becoming increasingly prevalent among Indigenous women, contributing to the transgenerational nature of metabolic disease [8–10].

Despite the significantly higher burden of metabolic comorbidities such as diabetes mellitus, obesity, dyslipidaemia and cardiovascular disease among First Nations Peoples with MASLD/MASH, this patient population appears to have a similar pattern of progression to decompensated cirrhosis when compared to non-Indigenous Australians. Similarly, after adjusting for sociodemographic and clinical factors, mortality did not vary according to Indigenous status. This finding should be interpreted with caution however, as it is likely that the significantly younger age of the First Nations cohort, and the median of 4.6 years of follow-up accruing a relatively small number of study end points affects these results. In fact previous studies of all-cause cirrhosis and hepatocellular carcinoma showed that First Nations Peoples were younger, had higher burden of comorbidities, higher rates of hospitalisation for cirrhosis, and poorer survival compared to non-Indigenous Australians [3, 4]. Moreover, as age is a key marker for duration of metabolic dysfunction and liver disease, and older age is an independent predictor of poorer survival, the significantly younger age of First Nations Peoples may be a key explanation for the findings. While we adjusted for age group in multivariable analysis, the data available for analysis were categorized and since the age cut-offs are arbitrary, incomplete adjustment for age is possible as the variation of a risk within a category may be wide. Importantly, the influence of ancestry and prevalence of genetic variants that may impact on the severity of MASLD has not been examined in First Nations Peoples. Racial disparity in the prevalence of MASLD has been reported, with the highest frequency among Hispanics and Caucasians and the lowest among African Americans [25]. Genetic factors play a role in the pathophysiology of MASLD, and the prevalence of genetic variations is influenced by ethnicity, affecting the susceptibility of different races to MASLD [26]. There is a paucity of genomic data for Aboriginal Australians, however the few genome-wide studies to date suggest there is a genetic predisposition towards obesity and diabetes in this population group, with similar archaic haplotypes being identified in Indigenous Australians as in other currently marginalised Indigenous groups [11, 12]. Over the years, observations have been made regarding the contribution of colonisation on the metabolic health of Indigenous Australians, resulting in a shift away from their traditional diets to more Westernised lifestyles [12–14].

Not unexpectedly, the strongest factors associated with progression to decompensated cirrhosis were the presence of portal hypertension and compensated cirrhosis at index admission, and their prevalence was similar according to Indigenous status. Extrahepatic cancer was also independently associated with liver disease progression and mortality, although fewer First Nations Peoples had cancer. Previous studies have reported comparable [27] or slightly higher incidence [28] of cancers and younger age [4, 29] at diagnosis in First Nations Peoples compared to the general Australian population. The fewer number of extrahepatic cancers in First Nations Peoples included in the current study was explained by the younger age of this cohort when compared to non-Indigenous Australians with MASLD/MASH.

As over two-thirds of First Nations Peoples lived outside major city areas and in most disadvantaged areas, this study additionally highlights the importance of primary care management of MASLD in this patient population. Many challenges are faced in chronic disease management in the setting of limited resources and socioeconomic disadvantage that exist in this population. Indeed, lack of access to diagnostic care for First Nations Peoples residing in remote areas in the Northern Territory was associated with a low rate of HCC detection from ultrasound surveillance, with non-surveillance HCC diagnosed at an advanced stage and consequent poor prognosis [30]. The development of Aboriginal Community-Controlled Health Services (ACCHS) as early as 1971 led to the delivery of culturally appropriate, autonomous primary health care services that are governed and delivered by local Aboriginal Australian communities through a First Nations majority elected board of directors [15]. Non-invasive serum-based testing for liver fibrosis (e.g. FIB-4 test) complemented by point-of-care transient elastography, can be performed in remote communities and can accurately screen for advanced fibrosis/cirrhosis in populations at risk of disease, without the need for travel to a metropolitan liver centre [31]. To overcome the geographical barriers to best medical care, coupled with culturally appropriate health care services, First Nations Peoples residing in remote communities would benefit significantly from ready access to non-invasive serum-based testing for liver fibrosis.

There are several well-established evidence-based strategies that enable effective chronic disease management in Indigenous populations. These include a strong chronic disease workforce made up of Indigenous Health Workers (IHWs), who play a crucial role in facilitating culturally safe communication, as well as other chronic disease health professionals such as dedicated care coordinators. This workforce requires regular access to relevant training and upskilling in the management of MASLD and cardiometabolic risk factors. Facilitating equitable access to specialists with expertise in liver disease and metabolic health by incorporating outreach services and telemedicine into primary care is also essential [7, 16]. Beyond the health system, there is a need for interventions centred around addressing the social determinants of health including education, employment and availability of community support services.

This population-based study included all people who had at least one hospital admission with a recorded diagnosis of MASLD/MASH. Conducting this study in the state of Queensland, the third most populous state (20.8% of the Australian population), and the second largest population of First Nations Peoples (29.2% of First Nations Australians), allowed the inclusion of a large number of First Nations Peoples [32]. Variability in data capture in the hospital admissions dataset can potentially lead to misclassification of MASLD/MASH status, cofactors and comorbidities. A previous study has shown that identification of MASLD/MASH through ICD-10-AM codes in Queensland hospitals may underestimate its prevalence [33]. While Hagstrom et al. advocate the identification of metabolic syndrome components (diabetes, obesity, hypertension, or dyslipidemia) at or before a hospital admission with MASLD to improve sensitivity of capturing MASLD-related cirrhosis, the accuracy of ICD-10-AM codes for identification of these are varied (e.g. sensitivity 95.8% for diabetes, 54.7% for obesity) [33]. Importantly, we have no reason to believe that underestimation of MASLD prevalence in the study varied according to Indigenous status. Exclusion of patients with co-existing liver disease, in particular those that are more common in First Nations Peoples (e.g. chronic hepatitis B, alcohol related cirrhosis, alcohol use disorder) [12], together with a high specificity of ICD-10-AM codes for NAFLD/NASH [33] means that misclassification of MASLD/MASH diagnosis among those included is small. Lack of data on fibrosis stage (e.g. fibrosis scores) is an important limitation as liver fibrosis is a strong predictor of a patient’s prognosis [34, 35].

## Conclusions

The high prevalence of diabetes and the younger age of First Nations Peoples with MASLD raises concern about future risk of progressive liver disease and highlights the importance of early identification of MASLD. Further studies with larger numbers of First Nations Peoples with MASLD (in particular MASLD without cirrhosis), and with longer follow-up are required to better examine the progression of MASLD to decompensated cirrhosis and mortality in this patient group. Provision of culturally appropriate intervention to reduce disease progression is crucial, in parallel with the management of cardiometabolic comorbidities. Our findings from an indigenous population in a high-income country emphasize the need for further MASLD research in indigenous populations.

### Electronic supplementary material

Below is the link to the electronic supplementary material.

Supplementary Material 1

## Data Availability

No datasets were generated or analysed during the current study.

## Abbreviations

CCI: Charlson Comorbidity Index
CI: Confidence interval
HCC: Hepatocellular cancer
HR: Hazard ratio
ICD-10-AM: International Classification of Diseases 10th edition– Australian Modification
IQR: Interquartile range
LASSO: Least absolute shrinkage and selection operators
MASLD: Metabolic dysfunction-associated steatotic liver disease
MASH: Metabolic dysfunction-associated steatohepatitis
NAFLD: Non-alcoholic fatty liver disease
