# Supplementary material for: High prevalence of diabetes among young First Nations Peoples with metabolic dysfunction-associated steatotic liver disease: a population-based study in Australia
Source: Int J Equity Health. 2024 Apr 30;23:84. doi: 10.1186/s12939-024-02153-z (PMC11061954; doi:10.1186/s12939-024-02153-z)

**Supplementary Fig. 1**. Cumulative incidence of decompensated cirrhosis according to Indigenous status for male, female, 20–49 years and 50 years and older (Kaplan–Meier Analysis)


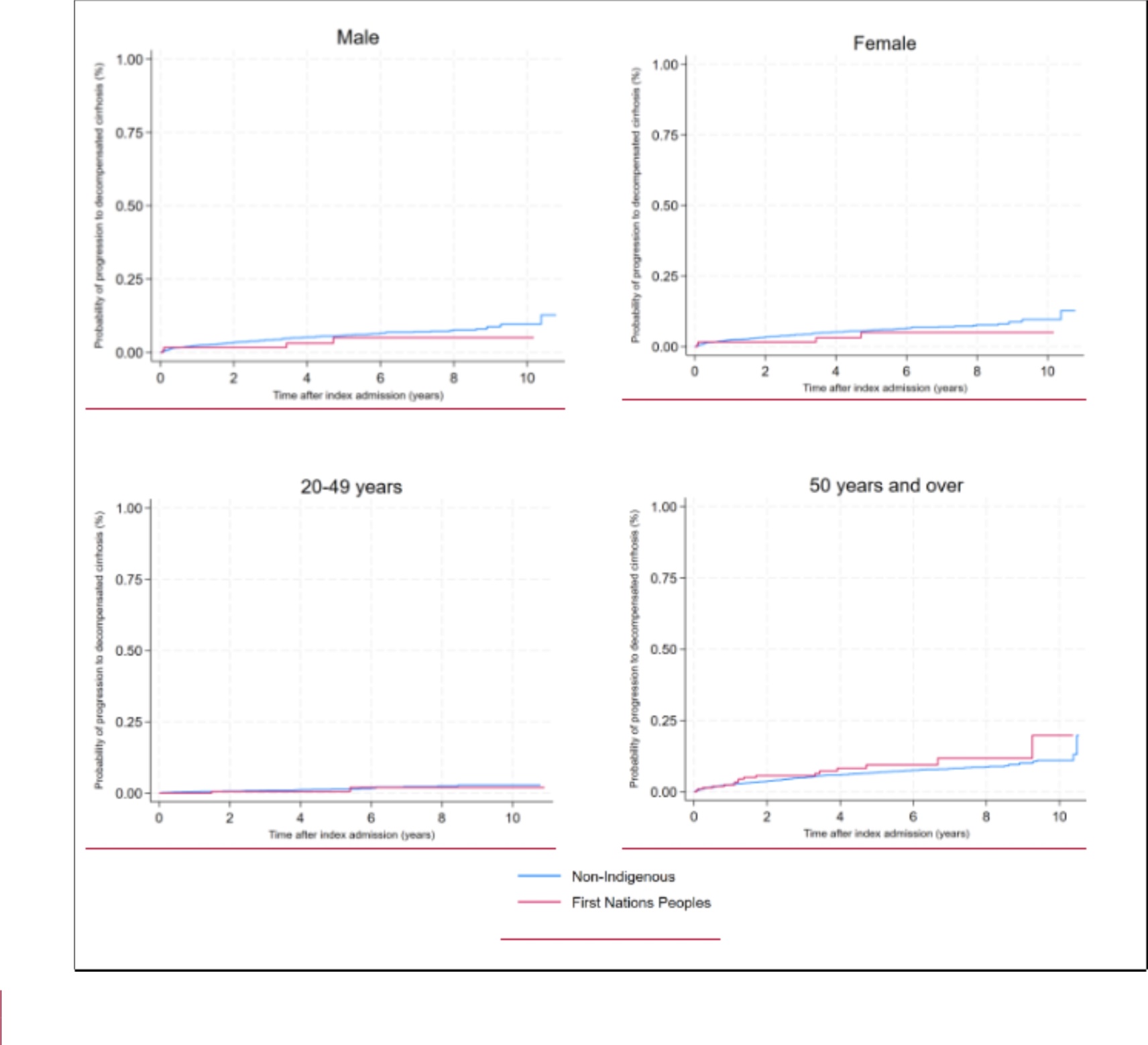


**Supplementary Fig. 2**. Kaplan Meier survival curve for overall mortality according to Indigenous status for male, female, 20–49 years and 50 years and older


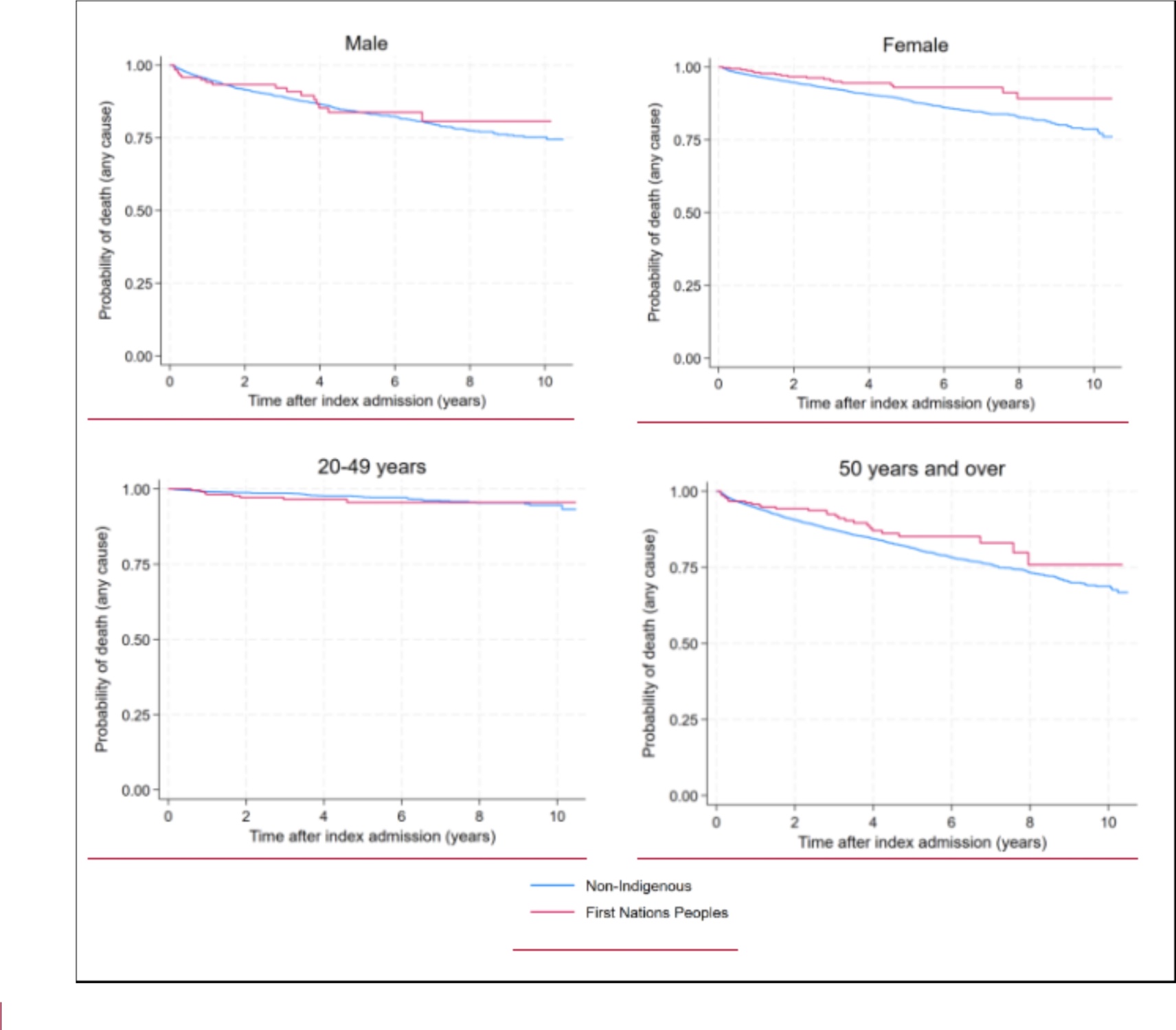

Supplement: Supplementary file 1 — Supplementary Material 1 [file 12939_2024_2153_MOESM1_ESM.docx]
